# Midazolam Reduces Metabolic Viability and Induces Apoptosis in MIA PaCa-2 Pancreatic Cancer Cells with Selective Transcriptional Modulation of Wnt and Hedgehog Components: A Preliminary In Vitro Study

**DOI:** 10.3390/ph19091472

**Published:** 2026-09-17

**Authors:** Irmak Fatoş Korkmaz, Tugba Elgun, Ciğdem Aktas, Sajjad Eslamkhah, Sevgi Kocyigit Sevinc, Asiye Gok Yurttas

**Affiliations:** 1 Department of Anaesthesia, University of Health Science, Bahçelievler State Hospital, Istanbul 34668, Türkiye; korkmazfatos1112@gmail.com; 2 Medical Biology, Faculty of Medicine, Biruni University, Istanbul 34015, Türkiye; telgun@biruni.edu.tr; 3 Biruni Advanced Technology Application and Research Center (B@MER), Biruni University, Istanbul 34015, Türkiye; mtaghizad@biruni.edu.tr; 4 Department of Genetics, Aziz Sancar Institute, Istanbul University, Istanbul 34452, Türkiye; cigdemaktas485@gmail.com; 5 Program of Pathology Laboratory Techniques, Vocational School, Biruni University, Istanbul 34015, Türkiye; 6 Department of Biophysics, Faculty of Medicine, Kutahya Health Sciences University, Kutahya 43100, Türkiye; sevgi.kocyigitsevinc@ksbu.edu.tr; 7 Department of Medical Biochemistry, Faculty of Medicine, Istanbul Atlas University, Istanbul 34403, Türkiye

**Keywords:** midazolam, MIA PaCa-2, pancreatic ductal adenocarcinoma, Wnt-associated transcriptional response, CTNNB1, apoptosis

## Abstract

**Background**. Pancreatic ductal adenocarcinoma remains difficult to treat and has an exceptionally poor prognosis. While prior studies have noted antitumour properties for midazolam in pancreatic cancer, whether this response involves developmental signaling pathways is unclear. Here, we evaluated midazolam-treated MIA PaCa-2 cells to identify associated Wnt and Hedgehog transcriptional shifts and their systems-level context. **Methods**. MIA PaCa-2 cells were exposed to midazolam (20–100 µM) for 24 h. We measured metabolic viability by MTT and apoptosis by Annexin V-FITC/7-AAD flow cytometry at 60 µM. Expression of 18 targeted genes was quantified by RT-qPCR using ACTB for normalization. Changed genes were examined through STRING/KEGG networks, TCGA-PAAD survival analysis, DGIdb annotations, and preliminary FZD7 molecular docking. **Results**. Midazolam reduced MTT-derived metabolic viability in a concentration-dependent manner (IC_50_ = 54.6 µM) and significantly increased Annexin V-positive apoptotic populations at 60 µM across three independent experiments. Eight transcripts changed significantly: CTNNB1, TGFBR2, WNT2, WNT7B, HHIP, and DHH declined, while CSNK1A1 and AXIN1 increased (all adjusted *p* < 0.05). Network analysis highlighted CTNNB1 as the main hub, and high CTNNB1 expression correlated with shorter survival in TCGA-PAAD patients (*p* = 0.043; HR = 1.58, 95% CI: 1.01–2.47). **Conclusions**. Midazolam reduced metabolic viability and increased apoptosis in MIA PaCa-2 cells alongside coordinated expression changes in key Wnt and Hedgehog components. Because the required doses far exceed clinical sedation levels and pathway activity was not directly confirmed at the protein level, these findings should be regarded as a preliminary hypothesis rather than evidence of therapeutic efficacy.

## 1. Introduction

Pancreatic cancer ranks among the leading causes of cancer-related mortality worldwide, and fewer than one in ten patients survives five years from diagnosis [1,2]. This outcome reflects late clinical presentation, early metastatic dissemination and pronounced resistance to conventional therapy [3,4,5]. Because most patients with resectable disease undergo surgery, and because the perioperative interval coincides with a period of heightened vulnerability to micrometastatic seeding, attention has turned to whether agents administered during anaesthesia influence tumour biology.

A body of experimental work indicates that anaesthetic drugs exert effects on tumour cells beyond their sedative and analgesic actions, modulating proliferation, apoptosis, migration and invasion [6,7,8]. Propofol has been reported to inhibit growth and induce apoptosis in pancreatic carcinoma through interference with PI3K/AKT and NF-κB signalling [9], and volatile agents such as sevoflurane have been associated with both pro- and anti-tumour effects depending on tumour type and experimental context.

Midazolam has attracted attention in this context. It has been reported to induce apoptosis in leukaemia and colon cancer cells through mitochondrial caspase-9 and caspase-3 activation with loss of mitochondrial membrane potential [10], to suppress proliferation and migration in lung carcinoma via EGFR/MEK/ERK signalling [11], and to inhibit proliferation and epithelial–mesenchymal transition in lung and breast cancer models [12]. Most directly relevant, Oshima and colleagues demonstrated antitumour and anti-inflammatory effects of midazolam in a mouse model of pancreatic ductal adenocarcinoma [13]. Mechanistically, midazolam acts principally as a positive allosteric modulator of the GABA_A receptor, and GABAergic signalling has itself been implicated in tumour biology: cancer-cell-derived GABA has been shown to promote β-catenin-mediated tumour growth and immunosuppression [14]. This provides a specific rationale for examining Wnt/β-catenin signalling as a candidate axis.

The Wnt/β-catenin pathway is frequently dysregulated in cancer and contributes to proliferation, epithelial–mesenchymal transition, stemness and apoptosis resistance [15,16], and its aberrant activation in pancreatic cancer has been linked to aggressive behaviour and therapeutic resistance [5,17]. Hedgehog signalling has likewise been implicated in pancreatic tumour progression and stromal remodelling, though with context-dependent and sometimes restraining effects [4,18]. Whether midazolam engages either axis in pancreatic cancer cells is unknown.

We therefore examined the effect of midazolam on MIA PaCa-2 cells, assessing MTT-derived metabolic viability, apoptosis and the expression of 18 targeted transcripts spanning Wnt/β-catenin, Hedgehog, proliferation and apoptosis-regulatory functions. Because antitumour effects of midazolam in PDAC have been reported previously, the incremental aim was to explore the associated targeted transcriptional pattern. Differentially expressed genes were additionally placed in context using PPI/KEGG analysis, a contextual TCGA-PAAD survival analysis, DGIdb interrogation and exploratory FZD7 docking. These in silico components were used solely for hypothesis generation and contextualisation and were not treated as independent validation of the experimental mechanism.

## 2. Results

### 2.1. Midazolam Reduces Metabolic Viability Concentration-Dependently

**Figure 1:**
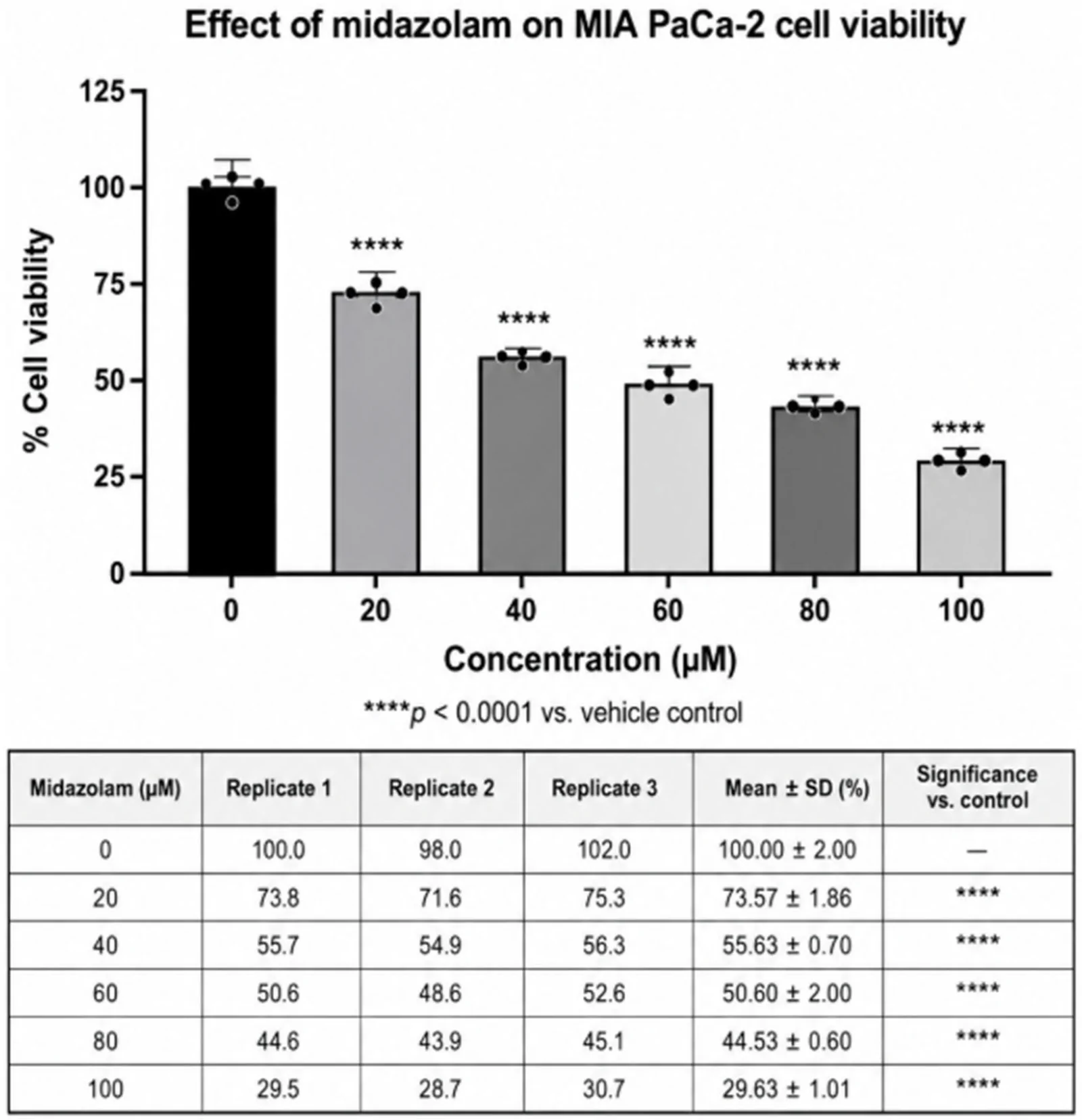
Effect of midazolam on MIA PaCa-2 cell viability. MIA PaCa-2 cells were treated with 0, 20, 40, 60, 80, and 100 µM midazolam for 24 h, and metabolic viability was assessed using the MTT assay. Data are presented as mean ± SD from three independent biological experiments, with individual replicate values displayed. Statistical comparisons between each midazolam concentration and vehicle control were performed using one-way ANOVA followed by Dunnett’s multiple-comparisons test. **** *p* < 0.0001 vs. vehicle control.

To evaluate the effect of midazolam on MTT-derived metabolic viability, MIA PaCa-2 cells were treated with 0, 20, 40, 60, 80, and 100 µM midazolam for 24 h. A concentration-dependent reduction in metabolic viability was observed (Figure 1). Because the MTT assay reflects cellular metabolic activity, these findings are interpreted as changes in metabolic viability rather than direct evidence of reduced proliferation or cytotoxicity.

Vehicle-treated cells showed a mean viability of 100.00 ± 2.00%. Midazolam reduced MTT-derived metabolic viability to 73.57 ± 1.86% at 20 µM, 55.63 ± 0.70% at 40 µM, 50.60 ± 2.00% at 60 µM, 44.53 ± 0.60% at 80 µM, and 29.63 ± 1.01% at 100 µM. One-way ANOVA demonstrated a significant overall effect of midazolam concentration (F(5,12) = 822.63, *p* < 0.0001), and Dunnett’s multiple-comparisons test showed that metabolic viability was significantly reduced at each tested midazolam concentration compared with vehicle control (all adjusted *p* < 0.0001). Individual values from the three independent biological experiments are displayed in Figure 1 together with the mean ± SD.

The concentration–response curve was analysed separately using a four-parameter logistic model, yielding an estimated IC_50_ of approximately 54.6 µM. These concentrations are suprapharmacological relative to typical plasma concentrations achieved during clinical sedation and should therefore not be interpreted as modelling routine perioperative exposure.

### 2.2. Midazolam Increases Annexin V-Positive Cell Populations at 60 µM

**Figure 2:**
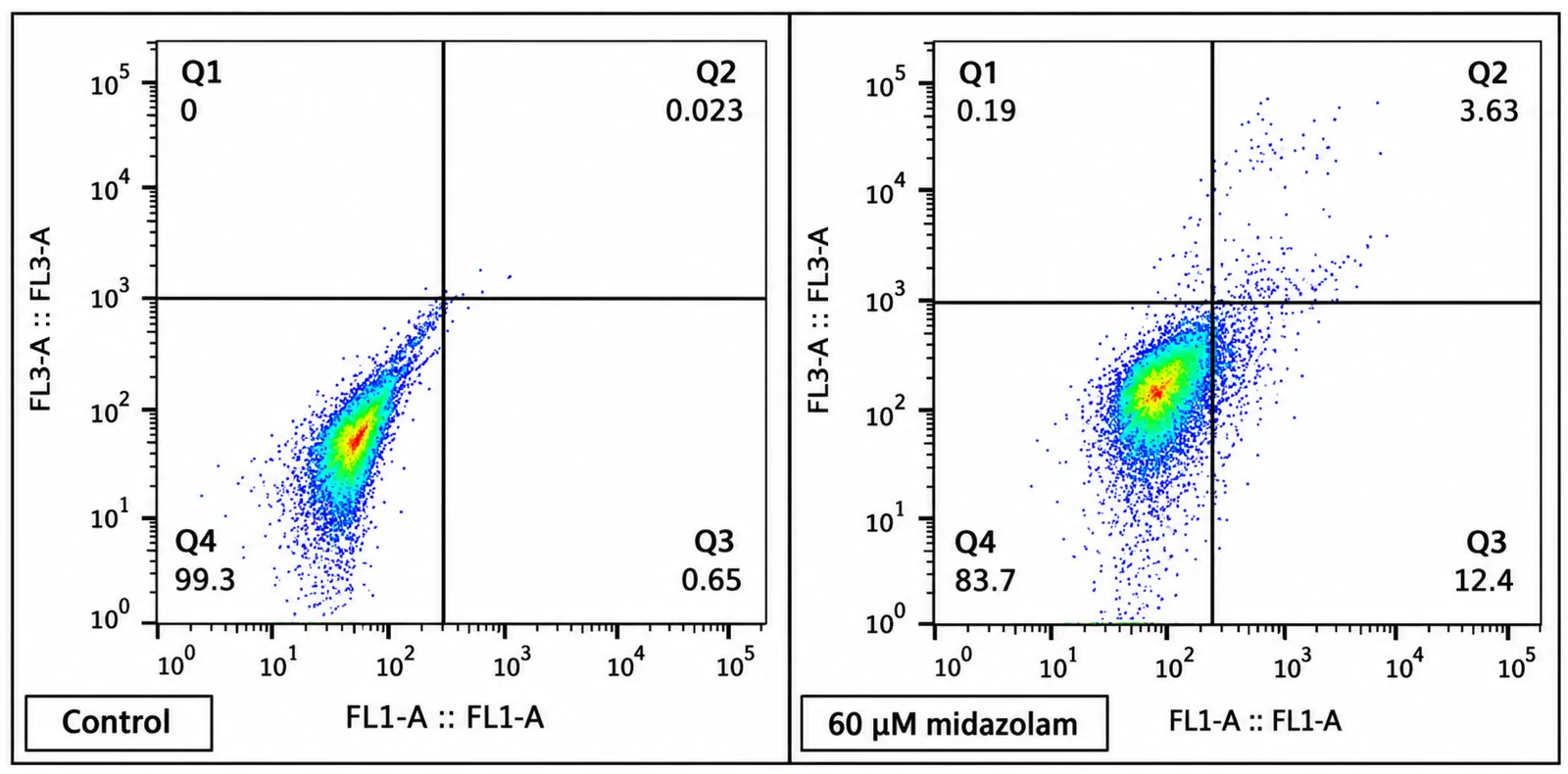
Flow-cytometric analysis of apoptosis in MIA PaCa-2 cells using Annexin V-FITC/7-AAD staining. Cells were treated with 60 µM midazolam for 24 h and compared with vehicle-control cells. Representative Annexin V-FITC/7-AAD plots. Q1: necrotic cells (Annexin V^−^/7-AAD^+^); Q2: late apoptotic cells (Annexin V^+^/7-AAD^+^); Q3: early apoptotic cells (Annexin V^+^/7-AAD^−^); Q4: viable cells (Annexin V^−^/7-AAD^−^).

To examine whether the reduced MTT-derived metabolic viability at approximately the IC_50_ was accompanied by apoptosis, MIA PaCa-2 cells were treated with 60 µM midazolam for 24 h and analysed by Annexin V-FITC/7-AAD double staining (Figure 2). Across three independent experiments, the viable cell fraction decreased from 99.30 ± 0.26% in vehicle-treated cells to 83.70 ± 0.30% following midazolam exposure (*p* = 0.00038). Early apoptosis increased from 0.650 ± 0.020% to 12.40 ± 0.60% (*p* = 0.00084), while late apoptosis increased from 0.023 ± 0.001% to 3.637 ± 0.060% (*p* = 0.000095). Consequently, the total apoptotic population increased from 0.673 ± 0.019% to 16.04 ± 0.66% (*p* = 0.00060). The necrotic population increased from 0.000 ± 0.000% to 0.190 ± 0.020% (*p* = 0.0037). Data are presented as mean ± SD from three independent experiments and were compared using two-tailed paired Student’s *t*-tests. Replicate-level values are provided in Supplementary Table S2.

### 2.3. Eight of Eighteen Transcripts Changed Significantly

**Figure 3:**
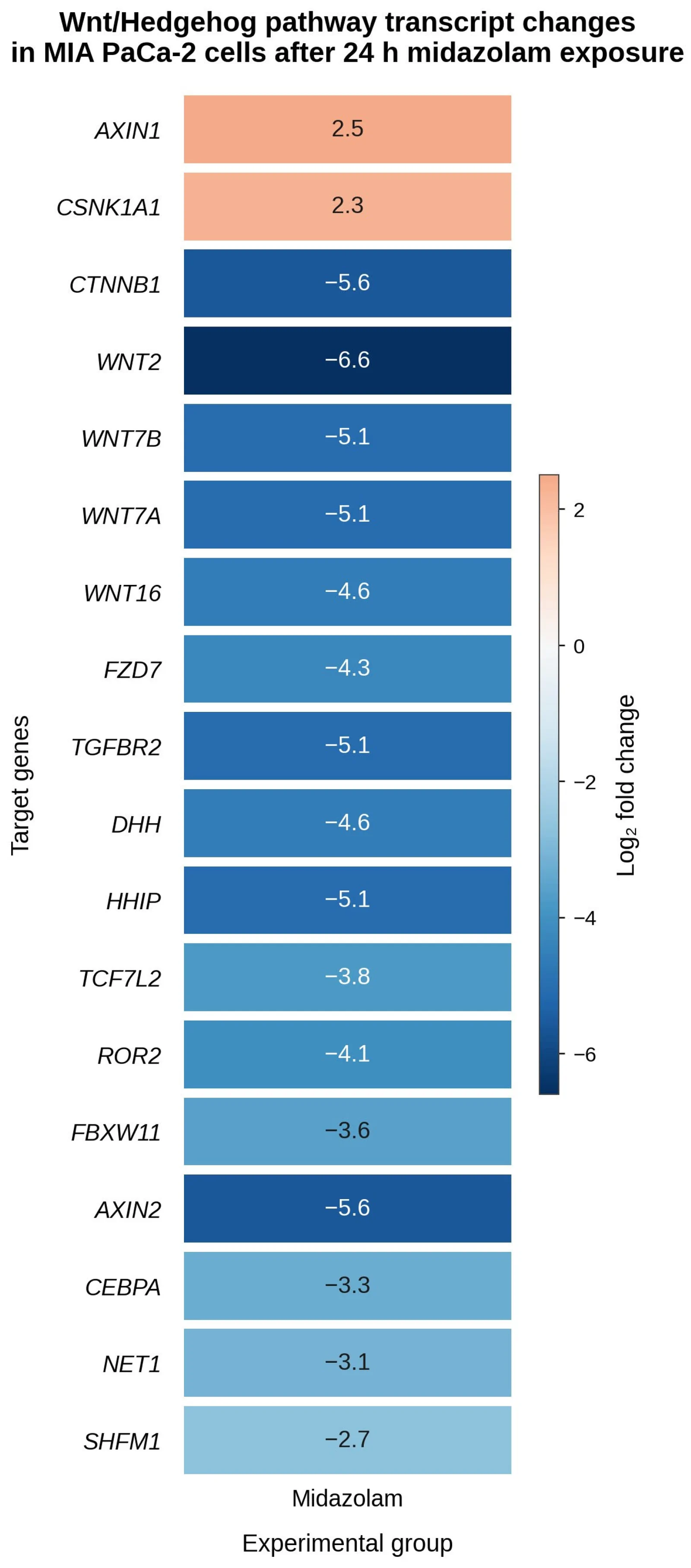
Targeted transcript changes in MIA PaCa-2 cells after 24 h midazolam exposure. Cells were exposed to 60 μM midazolam or vehicle for 24 h, and the expression of 18 targeted transcripts was quantified by RT-qPCR on a GeneQuery Human Basal Cell Carcinoma qPCR Array. Relative changes were calculated by the comparative 2^−ΔΔCq^ method using *ACTB* as the sole normalisation reference, and values are expressed as the mean log_2_ fold change relative to vehicle control; the control condition therefore equals 0 by definition and is not shown. Reactions were run in technical triplicate from three independent biological replicates (*n* = 3). The 18 genes are displayed in two blocks purely for layout; the sequence continues from the left block (*AXIN1*–*TGFBR2*) to the right block (*DHH*–*SHFM1*). Cell colour and the printed value both encode the log_2_ fold change (orange, increased; blue, decreased), and the colour scale spans the range covered by the data (−6.6 to +2.5). Statistical comparisons were performed on ΔCq values using two-tailed unpaired Student’s *t*-tests for each gene, with *p* values adjusted across the 18-gene panel by the Benjamini–Hochberg procedure. No fold-change threshold was imposed, so the ten unmarked transcripts are shown for completeness and should not be interpreted as changed.

Of the 18 targeted transcripts, eight were statistically significant after the stated multiple-testing procedure; no separate fold-change threshold was imposed. CTNNB1, TGFBR2, WNT2, WNT7B, HHIP and DHH were downregulated, while CSNK1A1 and AXIN1 were upregulated (Figure 3). The remaining ten transcripts were not statistically significant. The pattern is notable for its restriction. Within the Wnt module, two ligands and the central effector decreased while two destruction-complex components rose; within the Hedgehog module, a ligand and a pathway-regulatory protein decreased without accompanying change in downstream effectors.

### 2.4. Network and Pathway Analysis

**Figure 4:**
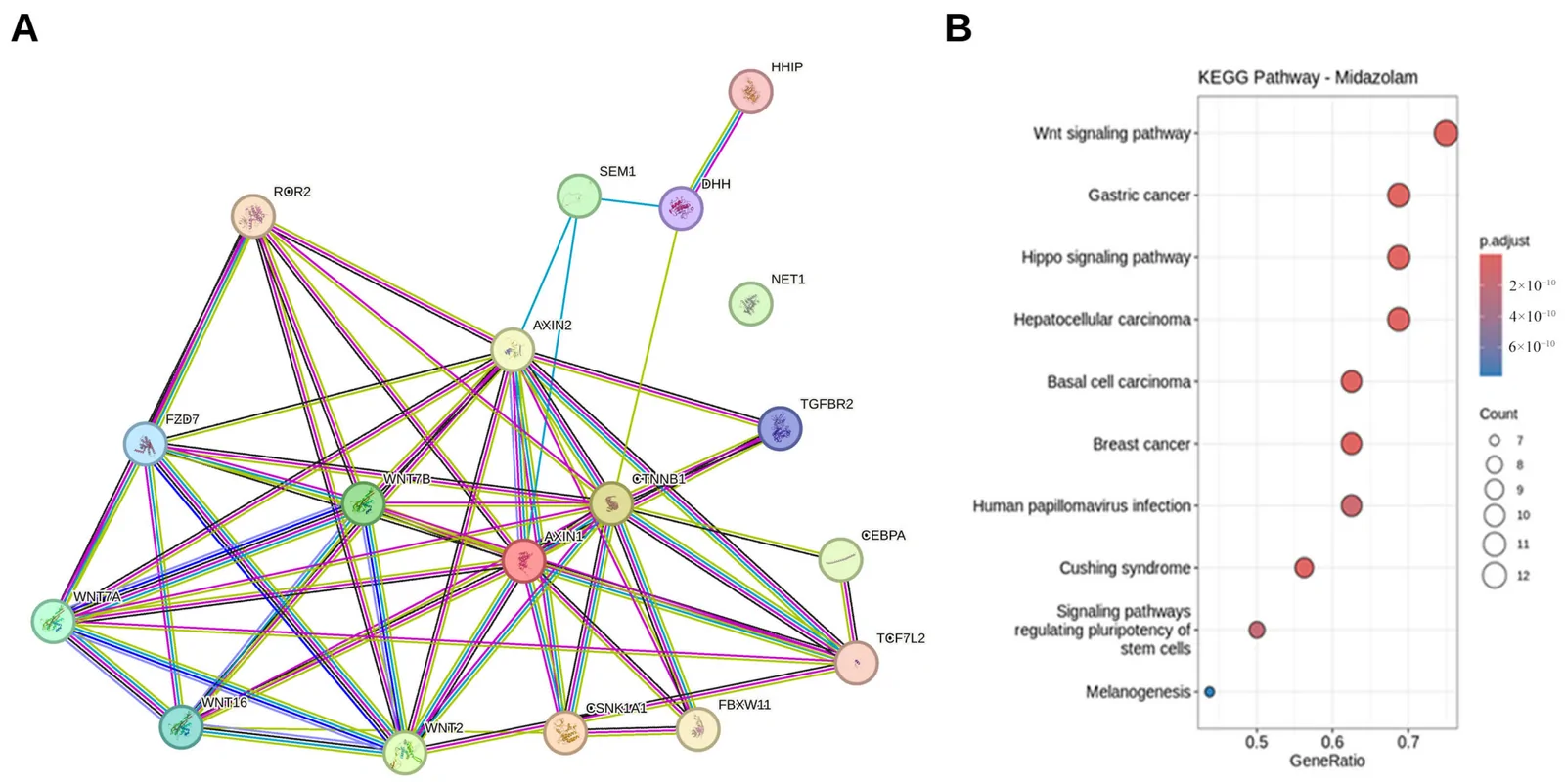
Exploratory systems-biology contextualisation of midazolam-associated transcript changes. (**A**) Protein–protein interaction network of the 18 targeted genes, retrieved from STRING v11.5 and visualised in Cytoscape [version 3.10.3], with *CTNNB1* identified as the highest-ranked hub by Maximal Clique Centrality. Each node represents one gene; node fill colours are assigned arbitrarily and carry no biological meaning. Edge colours denote the type of supporting evidence: light blue, curated databases; magenta, experimentally determined; green, gene neighbourhood; red, gene fusion; dark blue, gene co-occurrence; yellow-green, text mining; black, co-expression; lavender, protein homology. *SEM1* is the current HGNC symbol for the gene listed as *SHFM1* elsewhere in this work. (**B**) KEGG enrichment dot plot for the same input set. The *x*-axis shows the GeneRatio, dot size indicates the number of input genes annotated to each term (Count), and dot colour indicates the Benjamini–Hochberg-adjusted enrichment *p* value (*p*.adjust). Enrichment is descriptive and does not establish pathway upregulation or downregulation.

Exploratory protein–protein interaction mapping identified CTNNB1 as the highest-ranked hub by Maximal Clique Centrality (Figure 4A). KEGG annotation of the differentially expressed input set returned Wnt-related, Hippo, stem-cell pluripotency and several cancer-associated terms (Figure 4B). These results are descriptive of connectivity and annotation within a small, preselected targeted gene set and do not demonstrate pathway activation or suppression. The enrichment result requires particular caution because the input genes were drawn from a commercial panel enriched for Wnt/Hedgehog biology. Recovery of related pathways is therefore partly expected from panel composition and is not an independent validation of the experimental mechanism. We retain this analysis only as a systems-level consistency and contextualisation step.

### 2.5. Survival Association of CTNNB1 in TCGA-PAAD

**Figure 5:**
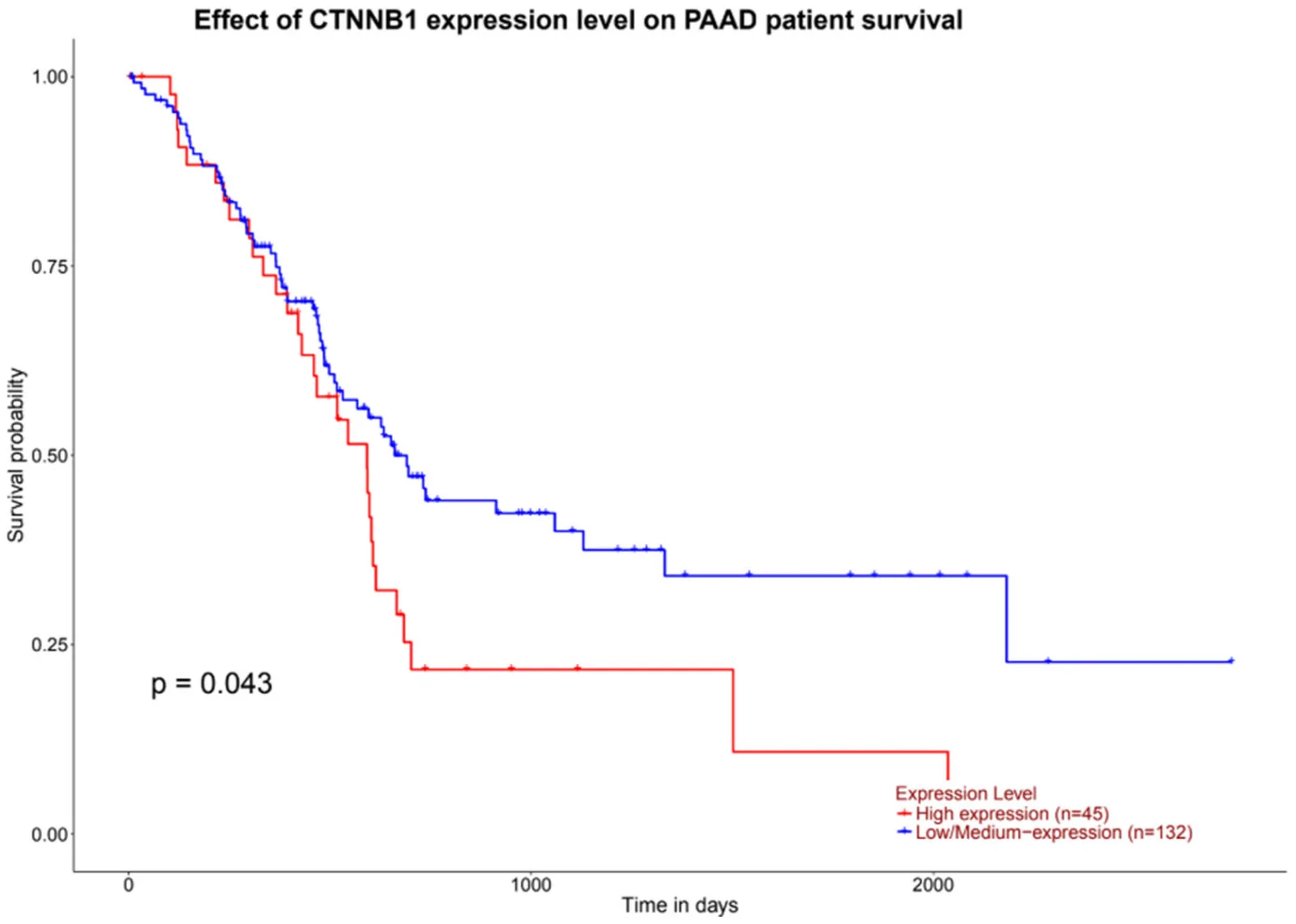
Overall survival analysis of CTNNB1 expression in TCGA-PAAD. Kaplan–Meier survival curves for patients with pancreatic adenocarcinoma stratified by a prespecified 75th-percentile (upper quartile) cutoff into high-expression (*n* = 45) and low/medium-expression (*n* = 132) cohorts. High *CTNNB1* expression was associated with significantly poorer survival (log-rank *p* = 0.043; Cox HR = 1.58, 95% CI: 1.01–2.47). Censored patients are marked with tick marks. The analysis provides retrospective disease-level context and does not imply mechanistic validation of the in vitro drug response.

In the TCGA-PAAD cohort (*n* = 177), patients with high CTNNB1 expression (*n* = 45, defined by the upper quartile) exhibited significantly shorter overall survival compared to the low/medium-expression group (*n* = 132; log-rank *p* = 0.043; Cox HR = 1.58, 95% CI: 1.01–2.47, *p* = 0.045) (Figure 5). Because this retrospective clinical association reflects endogenous CTNNB1 levels in untreated primary tumors, it does not demonstrate or validate the biological significance of midazolam-induced CTNNB1 downregulation observed in vitro. Instead, it provides disease-level context for the gene within pancreatic cancer biology.

### 2.6. Docking and Drug–Gene Interaction Analysis

**Figure 6:**
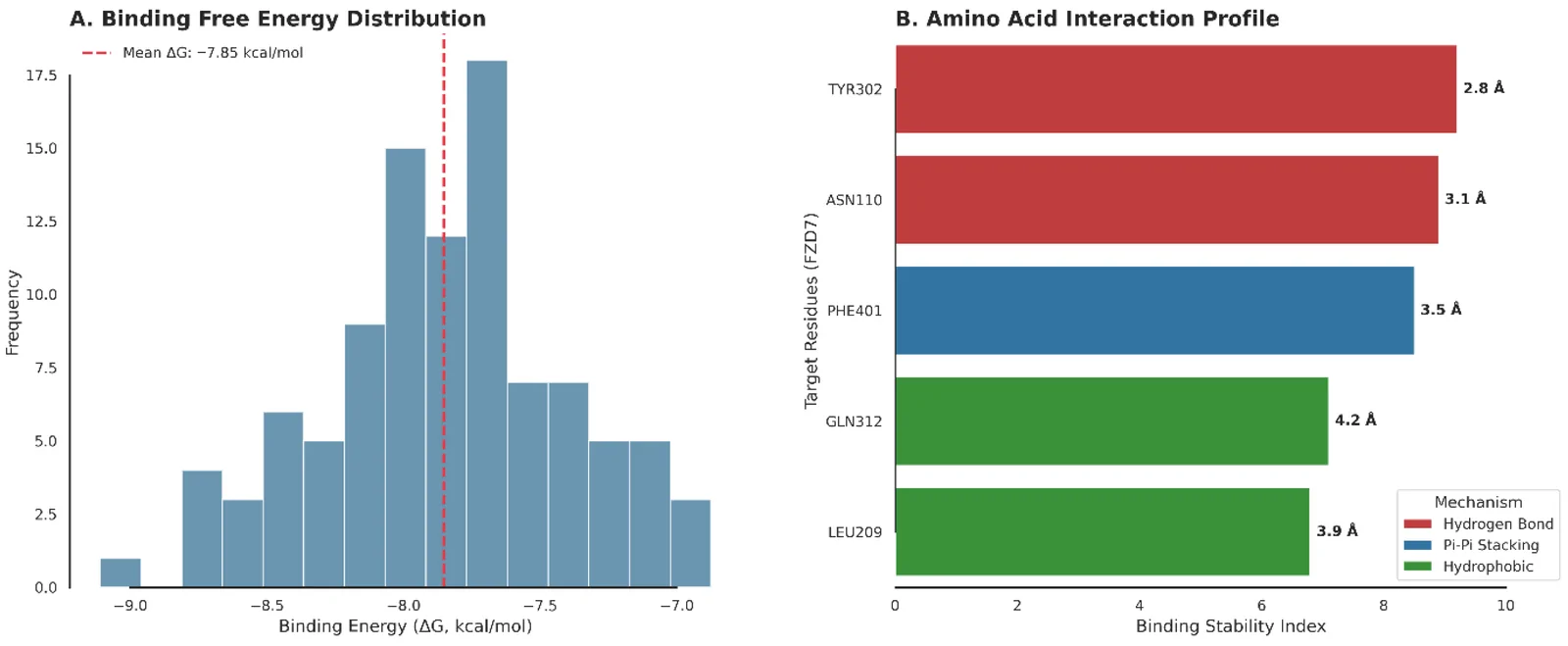
Exploratory in silico docking of midazolam to the Frizzled-7 (FZD7) cysteine-rich domain. (**A**) Distribution of predicted binding free energies across the docking poses; the dashed line marks the mean value of ΔG: −7.85 kcal/mol. (**B**) Predicted contacts between midazolam and individual FZD7 residues, with bar length indicating the binding stability index, the distance of each contact given in ångström, and bar colour denoting the interaction type (red, hydrogen bond; blue, π–π stacking; green, hydrophobic). The analysis illustrates predicted poses and contacts within the modelled lipid-binding region. The docking result is hypothesis-generating only and does not demonstrate direct FZD7 binding or establish FZD7 as a mediator of midazolam action.

Exploratory docking of midazolam to the FZD7 cysteine-rich domain returned a predicted binding-energy estimate of approximately −7.85 kcal/mol, with poses located within the modelled lipid-binding region (Figure 6A,B). Because FZD7 was selected as a Wnt-related structural candidate rather than established experimentally as a direct midazolam target, neither the docking score nor individual predicted contacts are interpreted as evidence of biologically relevant binding. Biochemical or biophysical target-engagement experiments would be required to test this hypothesis.

DGIdb querying returned several reported CTNNB1-associated drug–gene entries. This analysis is retained as exploratory pharmacological annotation of the CTNNB1 interaction neighbourhood (Table S1). It does not indicate that these compounds interact with midazolam, predict combination efficacy or synergy, or provide experimental validation of CTNNB1 as a mediator of the observed cellular response.

## 3. Discussion

The present study should be interpreted in the context of the more comprehensive work of Oshima et al. [13], who previously demonstrated antitumour and pro-apoptotic effects of midazolam in PDAC models, including in vivo experiments. Accordingly, our incremental contribution is not the discovery of an antitumour action of midazolam, but the observation of a restricted Wnt/Hedgehog-associated transcriptional pattern in MIA PaCa-2 cells that may inform future mechanistic studies. In our experiments, midazolam reduced MTT-derived metabolic viability concentration-dependently, and Annexin V/7-AAD analysis across three independent experiments showed a significant increase in apoptotic cell populations at 60 µM. The concentrations producing these effects—an IC_50_ in the region of 54.6 µM—exceed plasma concentrations attained during clinical sedation, which lie in the range of approximately 0.1 to 1 µM, by roughly two orders of magnitude. This is a central constraint on interpretation. The findings characterise an in vitro pharmacological response at suprapharmacological exposure; they do not indicate that perioperative sedation affects tumour biology, and no such inference should be drawn. Establishing potential clinical relevance would require demonstrating biological effects at concentrations approaching those achieved during clinical sedation and determining whether such effects are sustained with clinically relevant exposure durations [19,20,21,22].

The apoptotic profile is consistent with earlier reports in other tumour systems. Mishra and colleagues described midazolam-induced apoptosis in K562 leukaemia and HT-29 colon cancer cells proceeding through mitochondrial caspase-9 and caspase-3 activation with loss of membrane potential and DNA fragmentation [23], and Stevens and colleagues reported that midazolam activates the intrinsic apoptotic pathway independently of benzodiazepine and death receptor signalling [24]. We did not measure caspase activation, cytochrome c release or Bcl-2 family proteins, so the route of apoptosis in our model remains uncharacterised, and the mechanistic parallel is one of phenotype rather than of demonstrated mechanism.

The concurrent downregulation of CTNNB1, WNT2 and WNT7B alongside upregulation of AXIN1 and CSNK1A1 is internally coherent in one respect: reduced ligand and effector transcript abundance together with increased destruction-complex components would, taken at face value, predict reduced canonical Wnt output. Two considerations qualify this interpretation.

First, canonical Wnt activity is regulated predominantly at the level of β-catenin protein stability and nuclear localisation rather than CTNNB1 transcript abundance, and a change in mRNA carries no necessary implication for pathway output. Second, and more specifically, negative feedback within the pathway operates through AXIN2 rather than AXIN1: AXIN2 is a direct Wnt target gene and falls when signalling is suppressed, whereas AXIN1 is constitutively expressed and not a canonical target. Notably, AXIN2 was included in our panel but did not reach statistical significance (Section 2.3), precluding a definitive assessment of Wnt-specific feedback at the transcriptional level; this may reflect a true absence of change, insufficient statistical power, or both. Accordingly, the observed AXIN1 increase should not be interpreted as compensatory upregulation of a canonical Wnt feedback component. Direct assessment of active (non-phosphorylated) β-catenin and canonical Wnt output using a TOPFlash/FOPFlash reporter assay would resolve pathway activity more directly than transcript-level inference alone.

The GABAergic connection offers a plausible but untested framework. Midazolam acts principally through positive allosteric modulation of GABA_A receptors, and cancer-cell-derived GABA has been shown to promote β-catenin-mediated tumour growth and immunosuppression [6,25,26], which would place GABA signalling upstream of the axis we observed to change. However, GABA_A receptor expression was not assessed in our model, no receptor antagonist control was included, and at the 60 µM concentration used here, off-target or non-classical benzodiazepine effects cannot be excluded [26,27]. The hypothesis is therefore worth considering but is not supported by the present data.

Changes in HHIP and DHH indicate that the transcriptional response extended beyond Wnt to Hedgehog components. Interpretation here is constrained on two grounds. Hedgehog signalling in pancreatic cancer operates substantially through the stroma, and its role is context-dependent, with stromal Hedgehog response shown in some models to restrain rather than promote progression [28,29]. Cell-autonomous Hedgehog activity in a monoculture is correspondingly limited. Further, since HHIP and DHH were present on the array by virtue of its basal cell carcinoma design, their appearance among the changed genes reflects panel composition as much as biological selection. Confirmation of SHH, PTCH1, SMO and GLI1 on an independent platform is required before these transcripts are interpreted.

The TCGA-PAAD analysis provides disease-level context for CTNNB1, showing that high expression correlates with poorer overall survival (HR = 1.58, 95% CI: 1.01–2.47). However, endogenous CTNNB1 levels in untreated patient tumors cannot be causally linked to or validate the biological significance of midazolam-induced transcriptional downregulation in vitro. Similarly, PPI and KEGG pathway annotations are inherently shaped by the preselected composition of our targeted qPCR panel, DGIdb queries serve strictly as descriptive pharmacological annotations, and the exploratory FZD7 docking represents a hypothesis-generating structural observation lacking biophysical target-engagement confirmation. These computational evaluations are therefore retained solely as exploratory context and remain separated from our experimentally supported conclusions.

### Limitations

The concentrations required to elicit the reported effects exceed clinically attainable plasma levels by roughly two orders of magnitude, which represents the primary limitation of our findings. Because testing was not conducted in the 0.1–10 µM range, potential cellular responses at physiological exposure remain unaddressed.

All mechanistic observations were derived strictly from mRNA quantification without validation at the protein or functional level. In the absence of total/phosphorylated β-catenin, AXIN1, GLI1, or caspase measurements, as well as pathway-specific reporter assays, the proposed signaling links remain hypothetical.

Our experiments were restricted to MIA PaCa-2 cells, which harbor KRAS and TP53 mutations and display a mesenchymal phenotype. Consequently, this model does not reflect the genetic and stromal heterogeneity of pancreatic ductal adenocarcinoma. Furthermore, without testing against non-malignant pancreatic epithelial controls, tumor selectivity could not be determined. Follow-up studies in alternative PDAC cell models are necessary to assess the generalizability of this transcriptional profile.

Apoptosis and gene expression were examined at a single concentration and timepoint, precluding assessment of dose dependency or temporal kinetics. Although the apoptosis analysis included three independent experiments, broader multi-dose and time-course studies will be required to characterize the death response fully.

The targeted panel was small and originally configured for basal cell carcinoma; unassayed pathways could not be captured, which inherently constrained the enrichment analysis. ACTB was used as the sole reference gene for RT-qPCR normalization, and its stability under the 60 µM midazolam exposure condition was not independently validated; this methodological limitation should be considered when interpreting the relative transcript changes. Additionally, the MTT assay tracks metabolic activity and may reflect metabolic shifts rather than direct changes in cell viability or proliferation.

The bioinformatic and structural models should be viewed as exploratory. Network connectivity and pathway enrichments largely stem from the preselected nature of the array. The TCGA-PAAD survival assessment, based on an upper-quartile CTNNB1 threshold in treatment-naïve tumor samples, offers patient-level context but cannot be taken as functional confirmation of midazolam-induced transcriptional downregulation. Similarly, DGIdb annotations and FZD7 docking provide structural and pharmacological hypotheses rather than verified target binding.

## 4. Materials and Methods

### 4.1. Cell Line and Culture

MIA PaCa-2 human pancreatic adenocarcinoma cells (ATCC, Manassas, VA, USA) were maintained in RPMI-1640 (Gibco, Grand Island, NY, USA) with 10% fetal bovine serum and 1% penicillin–streptomycin at 37 °C in a humidified 5% CO_2_ atmosphere. Cells were passaged at 80–90% confluence with 0.25% trypsin–EDTA and used between passages 5 and 15.

### 4.2. Drug Preparation and Treatment

Midazolam (Sigma-Aldrich, St. Louis, MO, USA) was dissolved in DMSO to prepare a stock solution and diluted in complete medium immediately before use to obtain final concentrations of 20, 40, 60, 80, and 100 µM. Final DMSO did not exceed 0.1% (v/v) in any condition, and vehicle controls received an equivalent volume. Cells were seeded and allowed to adhere overnight before a 24 h exposure.

### 4.3. Cell Viability

Cells were seeded in 96-well plates at 1 × 10^4^ cells/well, allowed to adhere overnight and treated for 24 h. MTT (100 µL, 0.4 mg/mL; Sigma-Aldrich) was added and incubated for 3 h at 37 °C; formazan was dissolved in 100 µL DMSO (Merck, Darmstadt, Germany), and absorbance was read at 550 nm on a BioTek microplate reader (Winooski, VT, USA). Viability was expressed relative to vehicle control, and concentration–response data were fitted to a logistic concentration–response model to derive the IC_50_.

### 4.4. Apoptosis Analysis

Apoptosis and necrosis were assessed after 24 h at 60 µM midazolam, corresponding approximately to the experimentally determined IC_50_, using an Annexin V-FITC/7-AAD kit (BioLegend, San Diego, CA, USA). Cells were seeded at 6 × 10^5^ cells/well, and both adherent and floating populations were harvested, washed and resuspended in binding buffer. Three independent biological experiments were performed, with vehicle-control and 60 µM midazolam conditions analysed in parallel within each experiment. At least 10,000 events per sample were acquired on a BD Accuri C6 Plus cytometer (BD Biosciences, San Jose, CA, USA). Quadrant boundaries were set using unstained and single-stained compensation controls.

### 4.5. RNA Isolation and RT-qPCR

For transcript analysis, cells were evaluated after 24 h exposure to 60 µM midazolam or vehicle control. Vehicle-control and midazolam-treated RT-qPCR samples were generated as independent biological replicates and were not designed as matched experimental pairs. Total RNA was isolated with the innuPREP RNA Mini Kit 2.0 (Analytik Jena, Jena, Germany) and quantified on a NanoDrop 2000 (Thermo Fisher Scientific, Wilmington, DE, USA). Reverse transcription was conducted using the OneScript Plus cDNA Synthesis Kit (Applied Biological Materials Inc. [abm], Richmond, BC, Canada). qPCR was performed on a Roche LightCycler 480 using the GeneQuery Human Basal Cell Carcinoma qPCR Array (ScienCell Research Laboratories, Carlsbad, CA, USA). This array was selected because it provides validated primer sets covering the Wnt ligand, Frizzled receptor, destruction-complex and Hedgehog modules of interest; it is used here as a targeted developmental-signalling panel and not as a tumour-type classifier. Melt-curve analysis confirmed single-product amplification, and amplification efficiencies are provided as Supplementary Material.

The array contains manufacturer-predefined controls, including five housekeeping genes (ACTB, GAPDH, LDHA, NONO, and PPIH), as well as genomic DNA, positive PCR, and no-template controls. Although the commercial array includes five housekeeping genes, ACTB was used as the sole reference gene for normalization in the present study. GAPDH, LDHA, NONO, and PPIH were retained as manufacturer-provided housekeeping/control genes for assay quality control and were not included in the normalization calculation. Relative changes in target-gene expression were calculated using the comparative 2^−ΔΔCq^ method with ACTB as the normalization reference.

### 4.6. Bioinformatic Analysis

Differentially expressed genes from the targeted qPCR panel were used for exploratory contextual analyses. A protein–protein interaction network was constructed using STRING v11.5 and visualised in Cytoscape (version 3.10.3) with hub genes identified by Maximal Clique Centrality; KEGG pathway annotations were evaluated descriptively for the input gene set. To provide disease-level context, RNA-sequencing profiles and corresponding overall survival data from the TCGA Pancreatic Adenocarcinoma (TCGA-PAAD) cohort were retrieved via the UCSC Xena platform. Patients with complete clinical records (*n* = 177) were stratified using a prespecified upper-quartile threshold (75th percentile), assigning cases above this cutoff to the high-expression group (*n* = 45) and the remainder to the low/medium-expression comparator cohort (*n* = 132). Survival curves were estimated using the Kaplan–Meier method and compared by log-rank test, with hazard ratios (HRs) and 95% confidence intervals (CIs) calculated via univariate Cox proportional hazards regression. This retrospective analysis was interpreted solely as disease context rather than mechanistic validation of our in vitro findings. Drug–gene associations for CTNNB1 were obtained from DGIdb as exploratory pharmacological annotations.

### 4.7. Molecular Docking

Exploratory docking was performed between midazolam and the Frizzled-7 cysteine-rich domain (FZD7-CRD) using the apo crystal structure (PDB ID: 5T44). The receptor was prepared by removing heteroatoms and adding polar hydrogens, and the search region was centred on the lipid-binding groove of the CRD. Poses were ranked according to predicted binding energy. The ligand-bound FZD7-CRD structure (PDB ID: 5URV) and its crystallographic C24 fatty acid were used as structural protocol references, and NSC654259 was docked under the same workflow as a reference compound. FZD7 was selected post hoc as a Wnt-related structural candidate and was not experimentally established as a midazolam target. A verified 5URV redocking RMSD and a complete parameter set were not available; therefore, the docking analysis is interpreted only as a hypothesis-generating structural illustration and is not presented as a validated binding protocol or evidence of direct receptor engagement.

### 4.8. Statistical Analysis

Analyses were performed using GraphPad Prism 10.6.1 (GraphPad Software, Boston, MA, USA). For MTT, flow-cytometry, and RT-qPCR experiments, summary data are presented as mean ± SD from three independent biological replicates. Cell viability across multiple concentrations was analysed by one-way ANOVA followed by Dunnett’s multiple-comparisons test, with each treatment concentration compared with the vehicle control. The concentration–response model was fitted separately and used only to estimate the IC_50_. For flow-cytometry analysis, vehicle-control and 60 µM midazolam values obtained within the same independent experiment were treated as paired observations and compared using a two-tailed paired Student’s *t*-test. For transcript analysis, statistical comparisons were performed on ΔCq values using two-tailed unpaired Student’s *t*-tests between treated and vehicle groups for each gene. The resulting *p* values across the 18-gene panel were adjusted for multiple testing using the Benjamini–Hochberg procedure. Adjusted *p* values < 0.05 were considered statistically significant; no separate fold-change threshold was imposed.

## 5. Conclusions

Midazolam reduced MTT-derived metabolic viability in MIA PaCa-2 cells and increased Annexin V-positive apoptotic populations at 60 µM across three independent experiments, alongside a restricted targeted transcriptional response involving Wnt- and Hedgehog-associated genes. PPI/KEGG mapping, contextual TCGA-PAAD survival analysis, DGIdb interrogation and FZD7 docking provide complementary hypothesis-generating context but do not independently validate pathway activity, direct drug–target binding or clinical relevance. Because the observed effects occurred at suprapharmacological concentrations and protein-level/functional pathway validation was not performed, the findings should be interpreted as preliminary and model-specific. Further studies at clinically relevant concentrations, in additional PDAC and non-malignant pancreatic models, together with expanded dose- and time-course apoptosis analyses, protein/reporter assays and target-engagement studies, are required.

## Data Availability

The original contributions presented in this study are included in the article/Supplementary Materials. Further inquiries can be directed to the corresponding author.

## Supplementary Materials

The following supporting information can be downloaded at: https://www.mdpi.com/article/10.3390/ph19091472/s1, Supplementary Table S1. Exploratory CTNNB1-associated drug–gene entries retrieved from DGIdb. The table lists database-reported compounds, approval status, and interaction scores. These entries are presented for pharmacological annotation only and do not imply functional interaction with midazolam, therapeutic synergy, or mechanistic validation. Supplementary Table S2. Replicate-level Annexin V-FITC/7-AAD flow-cytometry percentages from three independent biological experiments, including mean ± SD and paired statistical comparisons.
