# Supplementary material for: Midazolam Reduces Metabolic Viability and Induces Apoptosis in MIA PaCa-2 Pancreatic Cancer Cells with Selective Transcriptional Modulation of Wnt and Hedgehog Components: A Preliminary In Vitro Study
Source: Pharmaceuticals (Basel). 2026 Sep 17;19(9):1472. doi: 10.3390/ph19091472 (PMC13610002; doi:10.3390/ph19091472)
Supplement: Supplementary file 1 [file pharmaceuticals-19-01472-s001.zip › Table S1.pdf]

Supplementary Material S1:

Table S1. Exploratory CTNNB1-associated drug–gene entries retrieved from DGIdb. The table lists database-reported compounds, approval status, and interaction scores. These entries are presented for pharmacological annotation only and do not imply functional interaction with midazolam, therapeutic synergy, or mechanistic validation.

Drug Name	Approval Status	Interaction Score
**CEQ508**	Not Approved	2.372900
**Fluorescein Sodium**	Approved	1.581933
**Eosin Y**	Not Approved	1.581933
**CHEMBL:CHEMBL2172378**	Not Approved	1.581933
**NMS-P715**	Not Approved	0.790967
**MW150**	Not Approved	0.790967
**Compound 12 [PMID: 22521646]**	Not Approved	0.790967
**BMS-582949**	Not Approved	0.790967
**NDPPC**	Not Approved	0.790967
**Recombinant Human Endostatin**	Not Approved	0.790967
