# Supplementary material for: Midazolam Reduces Metabolic Viability and Induces Apoptosis in MIA PaCa-2 Pancreatic Cancer Cells with Selective Transcriptional Modulation of Wnt and Hedgehog Components: A Preliminary In Vitro Study
Source: Pharmaceuticals (Basel). 2026 Sep 17;19(9):1472. doi: 10.3390/ph19091472 (PMC13610002; doi:10.3390/ph19091472)
Supplement: Supplementary file 1 [file pharmaceuticals-19-01472-s001.zip › Table S2.pdf]

Supplementary Material S2:

Supplementary **Table S2**. Replicate-level Annexin V-FITC/7-AAD flow-cytometry percentages from three independent biological experiments, including mean ± SD and paired statistical comparisons.

**Cell population**	**Phenotype**	**Condition**	**Experiment 1**	**Experiment 2**	**Experiment 3**	**Mean ± SD**	**Paired p value**
Viable cells	Annexin V^- / 7-AAD^-	Vehicle control	99.1	99.6	99.2	99.30 ± 0.26	
Viable cells	Annexin V^- / 7-AAD^-	60 µM Midazolam	83.7	83.4	84.0	83.70 ± 0.30	0.00038
Early apoptotic cells	Annexin V^+ / 7-AAD^-	Vehicle control	0.63	0.67	0.65	0.650 ± 0.020	
Early apoptotic cells	Annexin V^+ / 7-AAD^-	60 µM Midazolam	12.4	13	11.8	12.40 ± 0.60	0.00084
Late apoptotic cells	Annexin V^+ / 7-AAD^+	Vehicle control	0.024	0.022	0.023	0.023 ± 0.001	
Late apoptotic cells	Annexin V^+ / 7-AAD^+	60 µM Midazolam	3.63	3.7	3.58	3.637 ± 0.060	0.000095
Total apoptotic cells	Early + late apoptosis	Vehicle control	0.654	0.692	0.673	0.673 ± 0.019	
Total apoptotic cells	Early + late apoptosis	60 µM Midazolam	16.03	16.70	15.38	16.04 ± 0.66	0.00060
Necrotic cells	Annexin V^- / 7-AAD^+	Vehicle control	0.000	0.000	0.000	0.000 ± 0.000	
Necrotic cells	Annexin V^- / 7-AAD^+	60 µM Midazolam	0.19	0.17	0.21	0.190 ± 0.020	0.0037
